# Erythropoietin accelerates tumor growth through increase of erythropoietin receptor (EpoR) as well as by the stimulation of angiogenesis in DLD-1 and Ht-29 xenografts

**DOI:** 10.1007/s11010-016-2779-x

**Published:** 2016-08-20

**Authors:** A. Tankiewicz-Kwedlo, J. Hermanowicz, A. Surażynski, D. Rożkiewicz, A. Pryczynicz, T. Domaniewski, K. Pawlak, A. Kemona, D. Pawlak

**Affiliations:** 1 Department of Monitored Pharmacotherapy, Medical University of Bialystok, Mickiewicza 2C, Bialystok, Poland; 2 Department of Pharmacodynamics, Medical University of Bialystok, Mickiewicza 2C, Bialystok, Poland; 3 Department of Clinical Pharmacy, Medical University of Bialystok, Mickiewicza 2C, Bialystok, Poland; 4 Department of Medicinal Chemistry, Medical University of Bialystok, Mickiewicza 2C, Bialystok, Poland; 5 Department of Pathomorphology, Medical University of Bialystok, Mickiewicza 2C, Bialystok, Poland

**Keywords:** Erythropoietin, Erythropoietin receptor, Vascular endothelial growth factor receptor, Colon cancer

## Abstract

Anemia is a relatively common symptom coexisting with colorectal carcinoma. Besides having a positive impact on hematological parameters, erythropoietin (Epo) has the serious adverse effect of promoting the neoplastic process. The role of Epo in colon cancer has not been clearly shown. The aim of this study was to assess the effects of Epo therapy on colorectal carcinoma cells both in in vitro and in animal models. Human colon adenocarcinoma cells DLD-1 and Ht-29 were cultured in medium with Epo beta in normoxia. Cell proliferation was measured with an automated cell counter. Expression of erythropoietin receptor (EpoR) mRNA, Akt mRNA, and their proteins were assessed by RT-PCR and confocal microscopy, respectively. Nude mice were inoculated with adenocarcinoma cells and treated with a therapeutic dose of Epo. Expression of EpoR, VEGF, Flt-1 and CD31 was evaluated in xenograft tumors. We identified that Epo through EpoR activates Akt, which promotes colon cancer cell growth and proliferation. Epo, and high levels of phosphorylated EpoR, directly accelerates tumor growth through its proliferative and proangiogenic effects. This study demonstrated that Epo had enhanced carcinogenesis through increase of EpoR and Flt-1 expression, and thereby contributed to tumor development. These results suggest that both EpoR-positive and EpoR-negative cancer cells could be regulated by exogenous Epo. However, an increased response to erythropoietin was observed in the EpoR-positive cells. Thus, erythropoietin increases the risk of tumor progression in colon cancer and should not be used to treat anemia in this type of cancer.

## Introduction

Erythropoietin (Epo) is a glycoprotein that stimulates erythrocyte production and maturation. It is composed of 166 amino acids of a molecular weight of about 34 kD (depending on carbohydrate content) and is mainly (80–90 %) produced by renal interstitial cells. By binding to Epo receptor (EpoR) on the surface of early erythropoiesis progenitors (BCF-U), Epo promotes their survival, proliferation, differentiation and protects cells from apoptosis initiated by the JAK/STAT, PI3K-Akt, and Ras/ERK signaling pathway [1].

Colorectal carcinoma is a tremendous social problem. It is the second largest, after lung cancer, threat of modern oncology. The growth rate of colorectal carcinoma incidence in Poland is the highest in Europe. Anemia is a relatively common symptom coexisting with malignancy. It is a significant clinical problem occurring in approximately 60–90 % of patients with cancer, and in nearly 30 % of anemia is severe or life-threatening. In the case of colorectal carcinoma, the mean incidence of anemia ranges from 10 % to as much as 60 % [2], and major causes include chronic bleeding, impaired intestinal transit, and malabsorption .

According to cancer patients, the biggest problem is not pain but chronic fatigue caused by anemia. The results published in a 2004 ECAS (European Cancer Anemia Survey) observational study clearly demonstrated a close relationship between hemoglobin concentrations and patient functioning. It also proved that effective treatment of anemia, assessed as an increase in hemoglobin concentrations in the blood, often leads to a spectacular improvement in quality of life for cancer patients.

The introduction of Epo nearly 20 years ago revolutionized the treatment of this complication. Besides normalization of hematological parameters (increase in the number of erythrocytes and hemoglobin concentration), erythropoietin leads to improved effectiveness of radiation and chemotherapy, and shortens hospitalization. Better tumor tissue oxygenation increases the effectiveness of treatment by the so-called oxygen effect. It also helps to avoid interruption or postponing of successive cycles of chemo- or radiotherapy, and lowers doses of anticancer drugs. Erythropoietin is also an alternative for the transfusion of red blood cell concentrates, a method burdened with a variety of serious adverse effects.

Nevertheless, contrary to reports indicating beneficial effects of Epo in patients with cancer, some of the available data do not support such an action of erythropoietin, and actually negate it [3, 4]. Recent studies indicate that the use of erythropoietin to treat anemia in patients with proliferative lesions, besides having positive effects on hematological parameters, has the serious adverse effect of promoting the neoplastic process [5, 6].

Epo and its cognate receptor were expressed on various tumor tissues [7, 8] and numerous malignant cell lines [6]. The Epo/EpoR system is involved in angiogenesis in normal (uterine endometrium) [9] and tumor tissues (human hepatocellular carcinoma) [10]. It has been documented that Epo could play a biological role in rhabdomyosarcomas since it promotes proliferation and prevents differentiation of myoblasts [11]. Another report indicates that the Epo signaling pathway is an important component in colon carcinogenesis [12]. As a pleiotropic growth factor, Epo acts in a tissue-protective manner and exerts an antiapoptotic effect on head and neck squamous cell carcinoma [13]. Erythropoietin also supports survival of endothelial cells [14], neurons subjected to hypoxia [15], and thereby greatly supports survival of tumor cells such as breast cancer [16], head and neck [13], and colon cancer cells [17]. Due to its proliferative, antiapoptotic and stimulating angiogenesis activity, use of erythropoietin in cancer patients is controversial.

Three placebo-controlled clinical trials have shown that Epo has a negative effect on survival in patients with metastatic breast cancer [18], head and neck cancer [19], and lung cancer [20]. Because so many patients with colon cancer receive recombinant human Epo due to anemia, it is necessary to better understand the role of Epo in this particular tumor. At present, the effect of Epo on colon cancer cells is still unclear and poorly defined. There is no conclusive data describing the effect of Epo on colon cancer cells, which begs the question whether Epo has a direct impact on the progression of this cancer. Therefore, the purpose of the present study was to assess the effects of Epo therapy on colorectal carcinoma cells both in in vitro and in an animal model. We explored the effect of exogenous erythropoietin on proliferation of both the colon cancer cells DLD-1 and Ht-29. We also evaluated gene expression of EpoR in in vitro and in vivo conditions. In the animal model, we examined the effect of Epo on the neoplastic process and development of vascularization in the tumor.

## Methods

### Cell cultures

DLD-1 (CCL-221) and Ht**-**29 **(**HTB-38), human colorectal adenocarcinoma cell lines, were obtained from the American Type Culture Collection (ATCC, Manassas, VA, USA). The results of our previous research [21] and data from the literature [22] indicate that the DLD-1 line contains the EpoR gene and protein, and histologically is the most similar to a primary tumor. Line Ht-29, in turn, is a negative control of the EpoR gene and is used to assess multidrug resistance, absorption of nutrients, and chemically induced differentiation of enterocytes [22]. Cell line DLD-1 was cultured in RPMI 1640 medium (Sigma, USA), line Ht-29 in McCoy’s 5a medium (Sigma, USA) supplemented with 10 % fetal calf serum (Sigma, USA), penicillin (50 IU, Sigma, USA), and streptomycin (50 µg/l, Sigma, USA), in an incubator with 5 % CO_2_ (normoxia), at a relative humidity of 95 %, at 37 °C (Heraeus, USA). The culture media was changed every 2 days. Cells were generally maintained in 75 cm^2^ flasks (Sarstedt, USA). However, for the experiments, cells were plated in 100-mm dishes (Sarstedt, USA) with 10 ml of medium. The control was medium with PBS only. For all the experiments, cells in the fifth to ninth passage were used.

### Exogenous erythropoietin (Epo) administration

Colon cancer cell lines DLD-1 and Ht-29 were incubated for 24 h with exogenous erythropoietin beta (NeoRecormon, Roche, Switzerland) at concentrations of 1, 10, and 100 IU/ml in normoxia condition (21 % O_2_). Epo concentrations were chosen based on data from the literature. Doses of 1 IU/ml and 10 IU/ml correspond to the daily and weekly human dose, commonly used in the clinic [23]. Epo at a concentration of 100 IU/ml is normally used in these types of experiments, widely accepted and often used by other research teams [24, 25].

### Cell count

Quantification of total cells was carried out with an automated cell counter (NucleoCounter^®^ NC3000, Chemometec, Denmark).

### *Quantitative*-*real*-*time*-*PCR (*QRT-PCR) analysis

Total RNA was isolated from frozen tissues with the Thermo Scientific GeneJET RNA Purification Kit (Thermo Scientific, Lithuania), according to the manufacturer’s instructions. Quantification and quality control of RNA was determined using the *Thermo* Scientific *NanoDrop* 2000 spectrophotometer. An aliquot of 1 µg of total RNA was reverse transcribed with the RevertAid™ First Stand cDNA Synthesis Kit (Fermentas, Canada), according to the manufacturer’s instructions. *Quantitative*-*real*-*time*-*PCR (*QRT-PCR) was performed using the Stratagene Mx3005P QPCR System (Agilent Technologies, USA) with the SG qPCR Master Mix (2x) **(**EURx, Poland). Each reaction was run in duplicate and contained 2 µl of cDNA template along with 0.3 µM primers in a final reaction volume of 25 µl. Cycling parameters were 95 °C for 10 min to activate DNA polymerase, then 40 cycles of 95 °C for 15 s and 60 °C for 30 s, with a final recording step of 72 °C for 25 s to prevent any primer-dimer formation. PCR reactions were checked by including no-RT-controls, by omission of templates, and by melting curve to ensure only a single product was amplified. Standard curves were generated employing a series of four dilutions of cDNA for each gene.

Primers were designed using PRIMER-BLAST (http://www.ncbi.nlm.nih.gov/tools/primer-blast) software. Primer sequences were (5′-3′ forward, reverse): GATGGGTGGAGTCGCGT, CAGAGTTAAAAGCAGCCCTGG (GAPDH), TCATCCTCGTGGTCATCCTG, GAAGAGGCCTTCAAACTCGC (EpoR), and CGACGTGGCTATTGTGAAGG, TTGAGGAGGAAGTAGCGTGG (Akt).

Relative quantification of gene expression was determined by comparison of values of Ct using the ΔΔ*C*_t_ method. All results were normalized to glyceraldehyde 3-phosphate dehydrogenase (GAPDH).

### Immunofluorescence

Cells were cultured in BD FalconTM 96-well black/clear bottom tissue culture plates optimized for imaging applications at 10,000 cells per well. After 24 h of incubation, cells were rinsed with PBS and fixed with 3.7 % formaldehyde solution at room temperature for 10 min. After fixation, cells were washed three times with PBS and permeabilized with 0.1 % Triton X-100 solution at room temperature for 5 min. Then, cells were washed twice with PBS, and non-specific binding was blocked by an addition of a 3 % FBS solution, and the cells were incubated at room temperature for 30 min. After that time, the cells were rinsed, incubated with rabbit polyclonal anti-p-EpoR [(Tyr 456)-R, Santa Cruz Biotechnology] and rabbit polyclonal anti-p-Akt1/2/3 [(Ser 473)-R, Santa Cruz Biotechnology] antibodies for 1 h at room temperature, washed three times with PBS and incubated with fluorescent (FITC) anti-rabbit secondary antibody for 60 min in the dark. After washing, nuclei were stained with Hoechst 33342 (2 lg/ml) and analyzed using confocal microscopy imaging.

### Confocal microscopy

Cells were imaged with a BD Pathway 855 confocal system using a 209 (0.75 NA) objective. Cell populations were analyzed for cytoplasmic fluorescence intensity. Images of FITC-labeled cells were acquired using a 488/10 excitation laser and a 515LP emission laser. Image analysis was performed using ImageJ v1.51 software (National Institute of Health, USA).

### Establishment of xenograft

Experiments were conducted on 4-week-old mice, males weighing 18–20 grams, inbred strain Cby.Cg-Foxn1nu/J (Jackson Laboratory, USA). This strain has a hair follicle defect (homozygous males) and defective development of the thymic epithelium (athymic) and is commonly used for inducing cancer. All animals were kept in individual cages in a room with a constant temperature and humidity as well as a 12-hour light cycle. They also had free access to food and water. After a 1-week acclimation period, the animals were randomized into two groups. The mice in the first group were injected subcutaneously on the dorsal side with 50 μl suspension containing 1 × 10^8^ DLD-1 cells in PBS; while the second group of mice with 50 μl suspension containing 1 × 10^8^ Ht-29 cells in PBS, according to the method described by Shinohara et al. [26]. In order to determine tumor volume by external caliper, the greatest longitudinal diameter (length) and the greatest transverse diameter (width) were measured. Tumor volumes based on caliper survey were calculated using the modified ellipsoidal formula: [27]

$$V = \frac{\pi }{6} f \left( {{\text{lenght }} \times {\text{width}}} \right)^{{\frac{3}{2}}} .$$

Each group was divided into two subgroups: receiving subcutaneous injections of erythropoietin (Epo) and receiving solvent (vehicle) attached to NeoRecormon, used for specimen preparation (control). When the tumors reached a diameter of about 5 mm, i.e., according to the literature the size suitable to conduct further phases of research [17], Epo administration was started at 600 IU/kg (i.e., the therapeutic dose used in humans), three times a week [28]. Subcutaneous injections of Epo were continued in weeks 2 and 3. In week 4, the injections were not performed.

### Immunohistochemistry

Formalin-fixed, paraffin-embedded tissue slides were cut on the microtome into sections with a thickness of 4 mm. The slides were deparaffinized in xylenes and hydrated in alcohol. The antigen for the antibody was exposed in a citrate buffer with a pH of 6.0 for 20 min at 97 °C, then 20 min at room temperature. Endogenous peroxidase was blocked by incubation with 3 % hydrogen peroxide. The sections were incubated with rabbit polyclonal anti-EpoR (C-20, Santa Cruz Biotechnology), rabbit polyclonal anti-VEGF (A-20, Santa Cruz Biotechnology), rabbit polyclonal anti-Flt-1 (C-17, Santa Cruz Biotechnology), and rabbit polyclonal anti-CD31 (M-20, Santa Cruz Biotechnology) antibodies for 2 h at room temperature. Biotinylated anti-rabbit antibody and avidin conjugated with horseradish peroxidase (LSAB Rabbit Immunocruz Staining System, Santa Cruz Biotechnology) was used as a detection system. The color reaction to the peroxidase was performed with DAB. Protein expression was assessed by two independent pathologists in a quantitative manner based on the percentage of positively stained cells.

### Microvessel staining and counting

Immunohistochemical staining for CD31 was carried out to evaluate tumor microvessel density (MVD) in tumor tissues according to the method described by Ghanem et al. [29]. The number of CD31 positive vessels was evaluated by counting any positively stained endothelial cells or endothelial cell clusters as a single, countable microvessel in a ×400 field (×40 objective and ×10 ocular) in the five areas with the highest vascular density. The mean of five counts was defined as the MVD for each case. MVD was carried out by two independent investigators. The results of the CD31 counts were expressed as a means (SDs).

### Statistical analysis

Shapiro–Wilk’s *W* test of normality was used for data distribution analysis. In all experiments, mean values for four–ten assays ± SD or median (minimum–maximum), depending on characteristic distribution, were calculated. In the case of normally distributed data, *t* test or two-way ANOVA with post hoc Tukey HSD test were used to assess the significance of differences between groups. For non-normally distributed data, the Mann–Whitney *U* test was used. Pearson correlation coefficient was used to evaluate correlations between the studied parameters. Calculations were performed using Statistica 12.5 software. The differences were deemed statistically significant when *p* < 0.05.

## Results

### Erythropoietin increases number of DLD-1 cells but not Ht-29

**Fig. 1:**
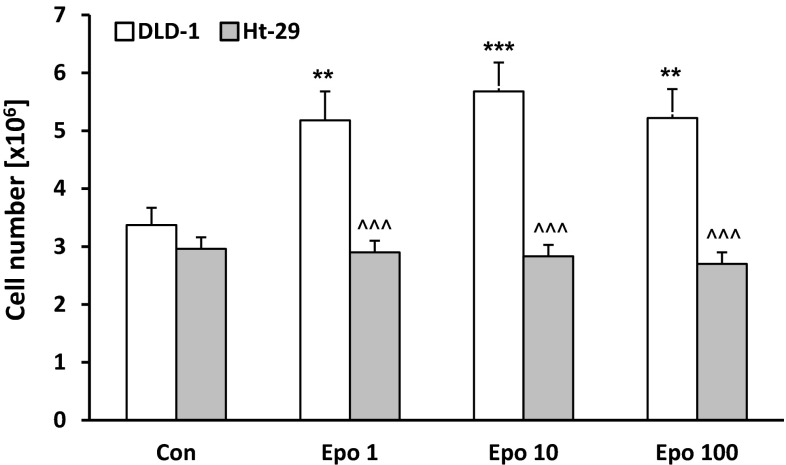
Number of DLD-1 and Ht-29 cells after 24-hour incubation with erythropoietin (Epo 1, 10, 100 IU/ml). Results are presented as means ± SDs, *n* = 6. **p < 0.01, ***p < 0.001 (Con in DLD-1 vs. Epo in DLD-1 cells); ^^^*p* < 0.001 (Epo in DLD-1 vs. Epo in Ht-29 cells)

The effect of erythropoietin on DLD-1 and Ht-29 numbers was measured with an automated cell counter after 24-hour incubation with increasing concentrations of erythropoietin. Measurements of cell numbers showed increased proliferation of DLD-1 cells after incubation with Epo (1, 10, 100 IU/ml) compared with the control group (*p* < 0.01, *p* < 0.001, *p* < 0.01, respectively) (Fig. 1). No differences in the number of cancer cells were found in Ht-29 cells growing with Epo compared with the control group. Significant differences in cell numbers between the two lines of cells incubated with Epo were observed (*p* < 0.001 for concentration of Epo 1, 10, and 100 IU/ml). Different effect on DLD-1 and Ht-29 cell numbers confirms the multidirectional and complex nature of Epo. It seems that erythropoietin has a weaker impact on cell line Ht-29.

### Erythropoietin increases p-EpoR level in EpoR-positive DLD-1, but does not impact the EpoR-negative Ht-29 colon cancer cell line

**Fig. 2:**
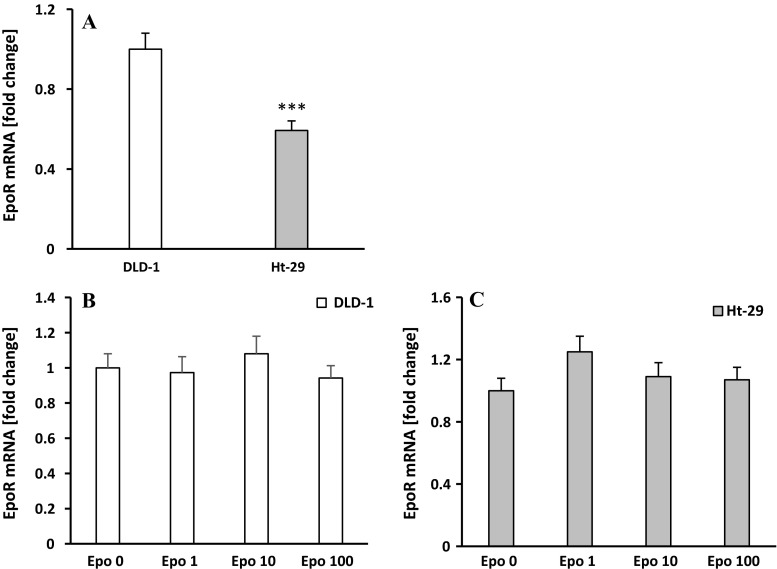
**a** Differences in EpoR mRNA expression between DLD-1 and Ht-29 line. EpoR mRNA levels in DLD-1 (**b**) and Ht-29 cells (**c**) after 24-hour incubation with erythropoietin (Epo 1, 10, 100 IU/ml). Results are presented as means ± SDs, *n* = 4. ***p < 0.05 (DLD-1 vs. Ht-2 cells)

Next, we wanted to confirm the dual nature of effect of erythropoietin on different colon cancer cell lines. RT-PCR analysis showed a significant increase of EpoR mRNA (*p* < 0.001) in DLD-1 cells compared with Ht-29 cells (Fig. 2a), which confirmed the literature data concerning the low expression of EpoR in line Ht-29. Administration of Epo in concentrations of 1, 10, and 100 IU did not change EpoR mRNA expression in both DLD-1 (Fig. 2b) and Ht-29 cells (Fig. 2c) after 24-h incubation compared with the control.

**Fig. 3:**
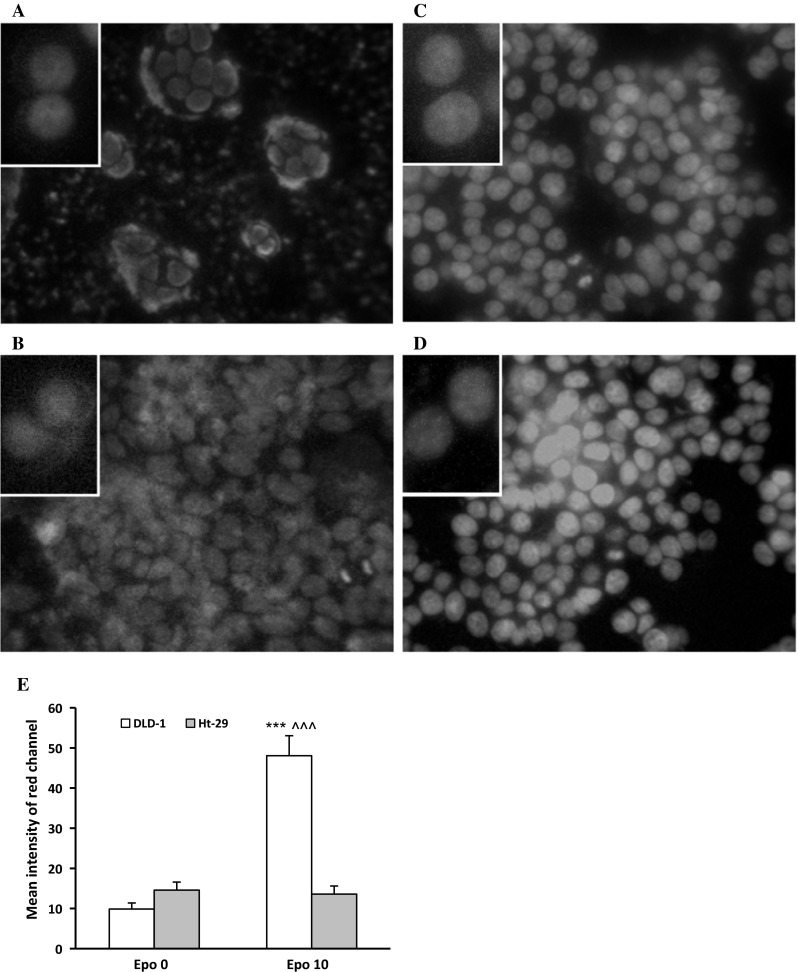
Phosphorylated EpoR levels in colon cancer cells. Confocal microscopy analysis of anti-phospho-EpoR (pEpoR, *red*) and the whole nuclei (Hoechst, *green*). Part **a** (without Epo, control) and **b** (with Epo) are DLD-1 cells. Part **c** (without Epo, control) and **d** (with Epo) are Ht-29 cells. **e** Quantitative analysis of confocal data. The *graph* shows mean intensity values of phosphorylated EpoR. Results are presented as means ± SDs, *n* = 4. ***p < 0.001 (Con in DLD-1 vs. Epo10 in DLD-1 cells), ^^^*p* < 0.001 (Con in DLD-1 vs. Con in Ht-29 cells). (Color figure online)

Confocal microscopy bio-imaging of phosphorylated EpoR (p-EpoR) confirmed differences in the presence of those receptors between cell lines (Fig. 3). Quantitative analysis of confocal data confirmed a significant increase in levels of p-EpoR in DLD-1 cells (*p* < 0.001) compared with Ht-29 cells (Fig. 3e). It also showed the localization of p-EpoR in DLD-1 membrane and cytoplasm (Fig. 3a, b). Clear assessment of the p-EpoR position was difficult in Ht-29 because of a low number of this receptor (Fig. 3c, d). Epo treatment increased level of p-EpoR in DLD-1 (Fig. 3b) but not in Ht-29 (Fig. 3d). Also quantitative analysis of confocal data confirmed a significant increase in levels of p-EpoR in DLD-1 cells compared with control (*p* < 0.001) and Ht-29 cells (*p* < 0.001) (Fig. 3e) after incubation with erythropoietin. Figure 3 presents unstimulated (a, c) and stimulated Epo (b, d) DLD-1 and Ht-29 cells, respectively, in which the whole nuclei (Hoechst, green) and the recruitment of the receptor (p-EpoR, red) were stained. Increased p-EpoR level in DLD-1 was a result of posttranslational modifications. This effect was not observed for the Ht-29 cells.

### Erythropoietin increases p-Akt level in DLD-1 and Ht-29 colon cancer cell lines

**Fig. 4:**
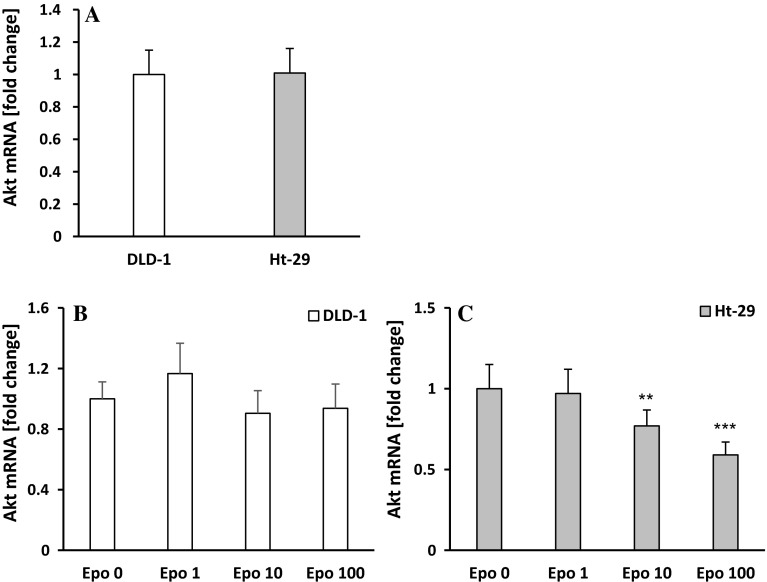
**a** Differences in Akt mRNA expression between DLD-1 and Ht-29 line. Akt mRNA levels in DLD-1 (**b**) and Ht-29 (**c**) cells after 24-hour incubation with erythropoietin (Epo 1, 10, 100 IU/ml). Results are presented as means ± SDs, *n* = 4. **p < 0.01 (Con in Ht-29 vs. Epo10 in Ht-29 cells), ***p < 0.001 (Con in Ht-29 vs. Epo100 in Ht-29 cells)

**Fig. 5:**
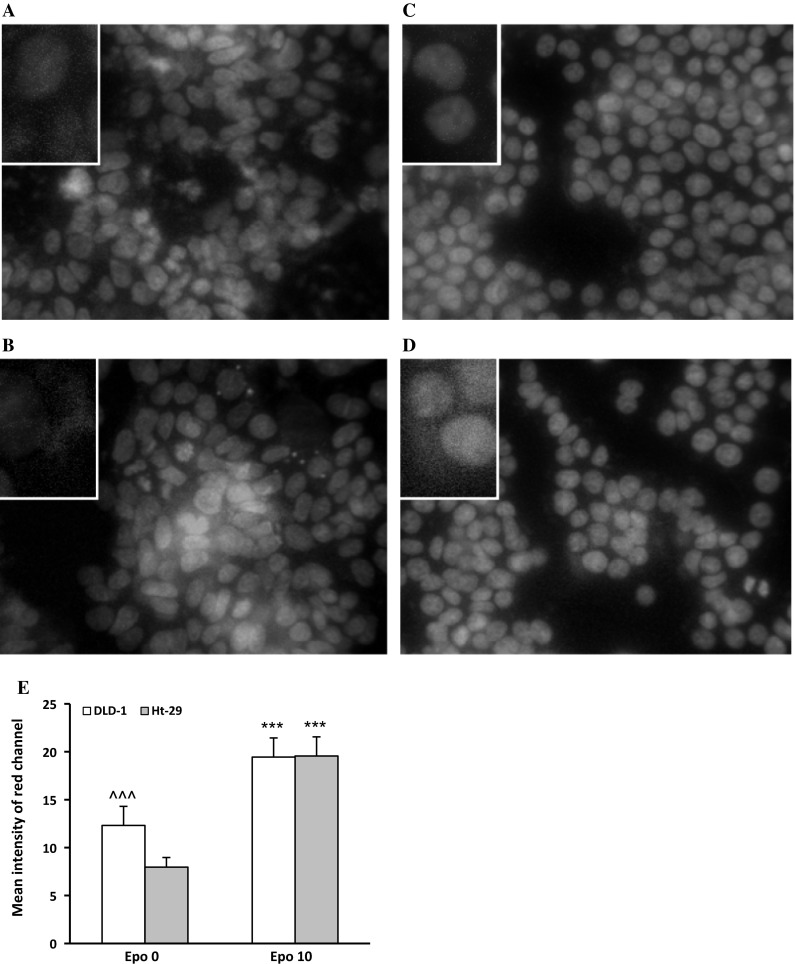
Phosphorylated Akt levels in colon cancer cells. Confocal microscopy analysis of anti-phospho-Akt (pAkt, *red*) and the whole nuclei (Hoechst, *green*). Part **a** (without Epo, control) and **b** (with Epo) are DLD-1 cells. Part **c** (without Epo, control) and **d** (with Epo) are Ht-29 cells. **e** Quantitative analysis of Confocal data. The *graph* shows mean intensity values of phosphorylated Akt, *n* = 4, ^^^*p* < 0.001 (Epo 0 in DLD-1 vs. Epo 0 in Ht-29 cells), ***p < 0.001 (Epo 0 in DLD-1 vs. Epo10 in DLD-1 cells; Epo 0 in Ht-29 vs. Epo10 in Ht-29 cells). (Color figure online)

We also evaluated the level of Akt, kinase that plays a key role in multiple cellular processes such as glucose metabolism, apoptosis, cell proliferation, transcription, and cell migration. Expression of Akt mRNA showed comparable levels in DLD-1 and Ht-29 cell lines (Fig. 4a). Epo (10,100 IU/ml) incubation did not change Akt mRNA expression in DLD-1 (Fig. 4b), but significantly decreased its level in Ht-29 cells (Fig. 4c), which presumably is associated with feedback loop in response to high level of p-Akt (*p* < 0.01, *p* < 0.001, respectively). High level of phosphorylated Akt (p-Akt) was indicated using confocal microscopy in DLD-1 and Ht-29 cells (Fig. 5). Figure 5 presents unstimulated (a, c) and stimulated Epo (b, d) DLD-1 and Ht-29 cells, respectively, in which the whole nuclei (Hoechst, green) and the recruitment of p-Akt (red) were stained. Quantitative analysis of confocal data showed a significant increase of p-Akt levels (*p* < 0.001) in DLD-1 cells compared with Ht-29 cells (Fig. 5e). Incubation with erythropoietin in concentration of 10 IU/ml increased levels of p-Akt in DLD-1 (*p* < 0.001) and Ht-29 cells (*p* < 0.001) (Fig. 5e). These results once again demonstrated the dualistic nature of erythropoietin on colon cancer cells.

### Erythropoietin stimulates tumor growth in a mouse xenograft tumor model

**Fig. 6:**
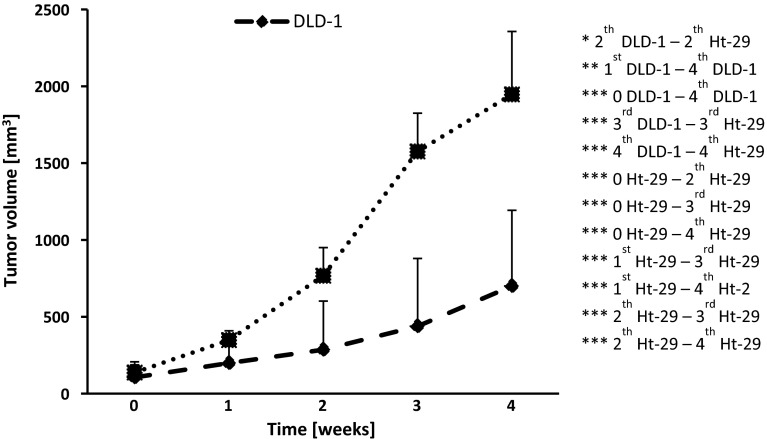
Dynamics of tumor growth in DLD-1 and Ht-29 xenografts. *0* start of observation, when the tumor was approx. 5 × 5 mm, *1* after the first week, *2* after the second week, *3* after the third week, *4* after the fourth week. Results are presented as means ± SDs, *n* = 10, * *p* < 0.05, **p < 0.01, ***p < 0.001

The main question at this point was whether the events observed in vitro had a counterpart in an in vivo model. To determine whether Epo can regulate tumor growth and proliferation in vivo, we injected subcutaneously DLD-1 and Ht-29 cells in athymic nude mice. Dynamics of tumor growth were higher in Ht-29 compared with DLD-1 xenografts (Fig. 6). In the second week of experiment, a significant difference in tumor volume in Ht-29 xenografts compared with DLD-1 xenografts (*p* < 0.05) and also with Ht-29 xenografts in 0 week (*p* < 0.001), in the third week (*p* < 0.001), with the fourth week (*p* < 0.001) was found. In the third week, in Ht-29 xenografts significant increase in tumor volume compared with 0 week (*p* < 0.001), with the first week (*p* < 0.001), as well with DLD-1 xenografts (*p* < 0.001) were observed. In the fourth week of the study, a significant difference in tumor volume in DLD-1 xenografts compared with 0 week (*p* < 0.001), with the first week (*p* < 0.01) and with the fourth week in Ht-29 xenografts (*p* < 0.001), and also in Ht-29 xenografts compared with 0 week (*p* < 0.001), with the first week (*p* < 0.001) was found.

**Fig. 7:**
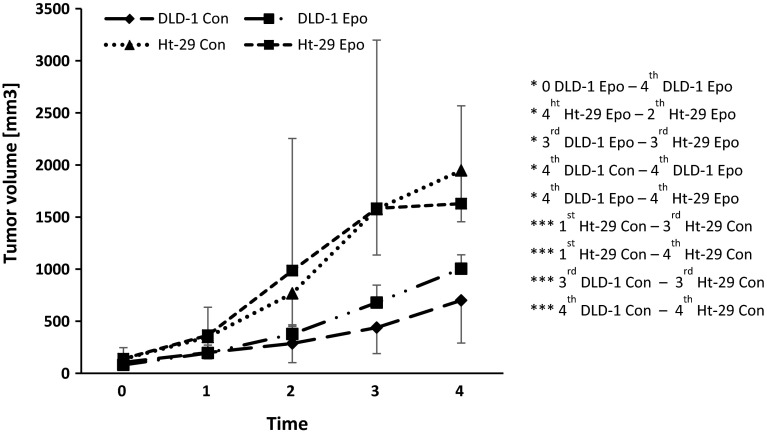
Effect of erythropoietin (Epo, 600 IU/kg) on tumor volume in DLD-1 and Ht-29 xenografts. *0* start of observation, when the tumor was approx. 5 × 5 mm, *1* after the first week, *2* after the second week, *3* after the third week, *4* after the fourth week. Results are presented as means ± SDs, *n* = 10. *p < 0.05; **p < 0.01; ***p < 0.001

Then, DLD-1 and Ht-29 cells were injected subcutaneously into a new group of athymic nude mice. In the third week of the experiment, a significant increase in tumor volume in control Ht-29 xenografts compared with control DLD-1 xenografts (*p* < 0.001) and control Ht-29 xenografts of the first week (*p* < 0.001) was observed. Epo administration for 3 weeks resulted in a significant increase in tumor volume in Ht-29 xenografts compared with DLD-1 xenografts (*p* < 0.05), which is associated with morphometric feature of Ht-29. Chronic administration of Epo resulted in a significant increase in tumor volume compared with the control (*p* < 0.05) in the fourth week, as well as compared with the value observed before drug administration (*p* < 0.05) in DLD-1 xenografts. In the fourth week, we noted a significant increase in tumor volume in control Ht-29 xenografts compared with control DLD-1 xenografts (*p* < 0.001) and Ht-29 xenografts of the first week of experiments (*p* < 0.001). We also found a significant increase in tumor volume in Ht-29 xenografts treated with Epo compared with DLD-1 xenografts receiving Epo (*p* < 0.05) as well as tumor volume in the second week in these groups (*p* < 0.05) (Fig. 7). The proportional volume changes were observed in Ht-29 xenografts during the experiment. In turn, the increase in tumor volume in DLD-1 xenografts was slower. Response to erythropoietin was stronger in DLD-1 xenografts, which confirms the involvement of EpoR in promoting the cancer process.

The most aggressive grade 3 was found in all DLD-1 xenografts (100 %). In the case of control Ht-29 xenografts, 40 % of individuals had grade 3, while other animals grade 2 (Table 1). Immunopathological study revealed faster growth of poorly differentiated cancer cells. In control Ht-29 xenografts mitotic index was higher compared with DLD-1 xenografts (*p* < 0.001), as confirmed by Mann–Whitney non-parametric *U* test. It may have led to rapid tumor growth in these animals. Immunohistochemical staining indicated an increase of mitotic index in DLD-1 xenografts treated with Epo compared with the control (*p* < 0.05). In Ht-29 xenografts treated with Epo, reduction of the mitotic index was observed compared with the control (*p* < 0.05). In EpoR-negative Ht-29 xenografts tumor development was slower as demonstrated by reduced mitotic index after Epo treatment. The results of these studies confirm the significant impact of erythropoietin mainly on EpoR-positive cells.

**Table 1:** Effect of erythropoietin (Epo, 600 IU/kg) on selected histopathological parameters in DLD-1 and Ht-29 xenografts

Group of xenografts	Grade (*n*, % of total)		Mitotic index median (min–max)	Necrosis (*n*, % of total)
	2	3		
DLD-1 Con	0/10 (0 %)	10/10 (100 %)	3.0 (2.0–8.0)	10/10 (100 %)
DLD-1 Epo	0/10 (0 %)	10/10 (100 %)	8.5 (2.0–13.0)*	10/10 (100 %)
Ht-29 Con	6/10 (60 %)	4/10 (40 %)	9.5 (4.0–17.0)^^^	9/10 (90 %)
Ht-29 Epo	0/10 (0 %)	10/10 (100 %)	4.5 (2.0–13.0)*	9/10 (90 %)

Results are presented as absolute values, percentages, and median with minimum and maximum values, *n* = 10

* *p* < 0.05 (Con in DLD-1 vs. Epo in DLD-1, Con in Ht-29 vs. Epo in Ht-29)

^^^ *p* < 0.001 (Con in DLD-1 vs. Con in Ht-29)

**Fig. 8:**
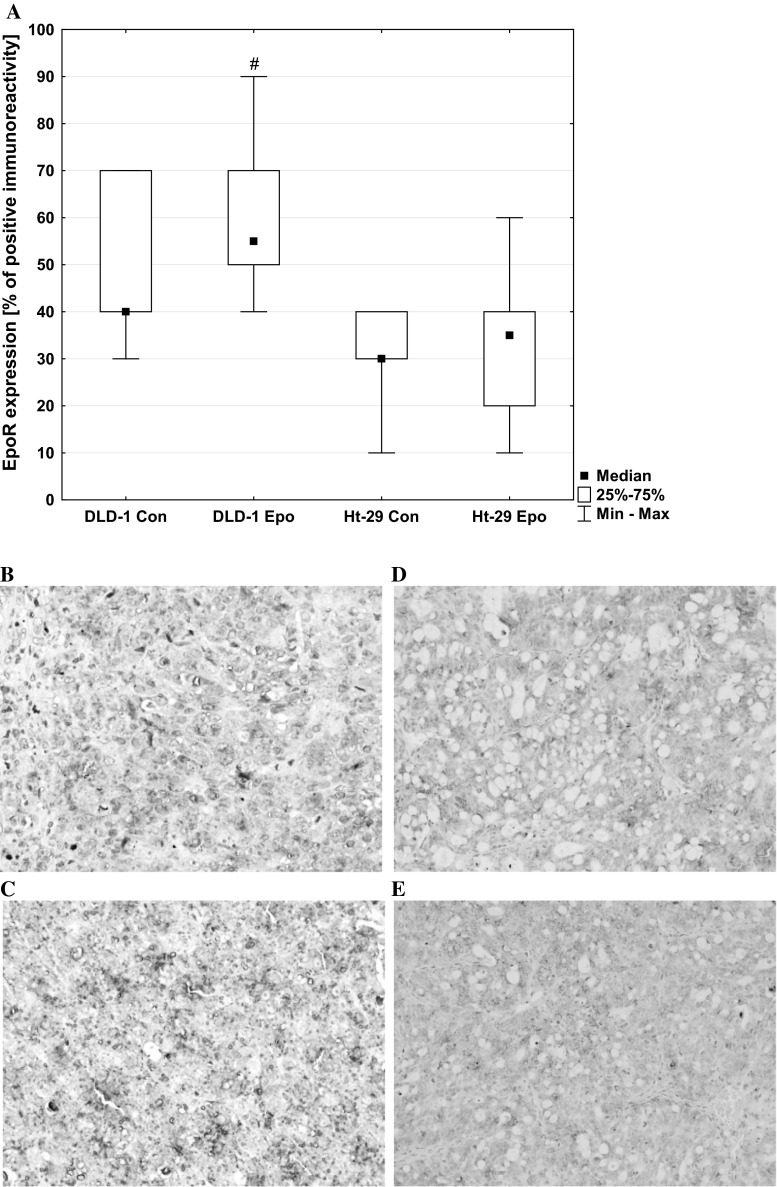
Positive expression of EpoR in membrane, cytoplasm, and intracellular localization of colon cancer xenografts: **a** a box-and-whisker plot of percent of EpoR expression in DLD-1 and Ht-29 tumor xenografts. Results are presented as medians (minimum–maximum), *n* = 10, #*p* < 0.05 (Epo in DLD-1 vs. Epo in Ht-29 xenografts). **b** expression in control DLD-1 xenografts; **c** expression in DLD-1 xenografts receiving Epo; **d** expression in control Ht-29 xenografts; **e** expression in Ht-29 xenografts receiving Epo (H&E staining; *magnification* ×400)

Microscopic observations showed necrosis in 100 % of DLD-1 and in 90 % of Ht-29 xenografts (Table 1). Cytoplasmic, membrane and intercellular reaction anti-EpoR staining prevailed in all positive slides. In the present study, positive immunoreactivity for EpoR was found in all DLD-1 xenografts: 7 (35 % of all cases) of these exhibited weak staining and 13 (65 % of all cases) exhibited strong staining. In Ht-29 xenografts, positive immunoreactivity for EpoR was observed also in all animals; however, only 1 (5 % of all cases) exhibited strong staining. In Ht-29 xenografts, weak immunoreactivity was found in 19 cases (95 % of positive tumors), while strong immunoreactivity was detected in 1 case (5 % of positive tumors). Mann–Whitney non-parametric *U* test confirmed the significant differences in EpoR expression in erythropoietin-treated DLD-1 xenografts compared with Ht-29 xenografts (*p* < 0.05) (Fig. 8). In control DLD-1 and Ht-29 xenografts, EpoR expression significant negatively correlated with tumor weight (*r* = −0.524, *p* < 0.05). Epo treatment abolished this correlation.

### Erythropoietin stimulates angiogenesis in EpoR-positive xenografts

**Fig. 9:**
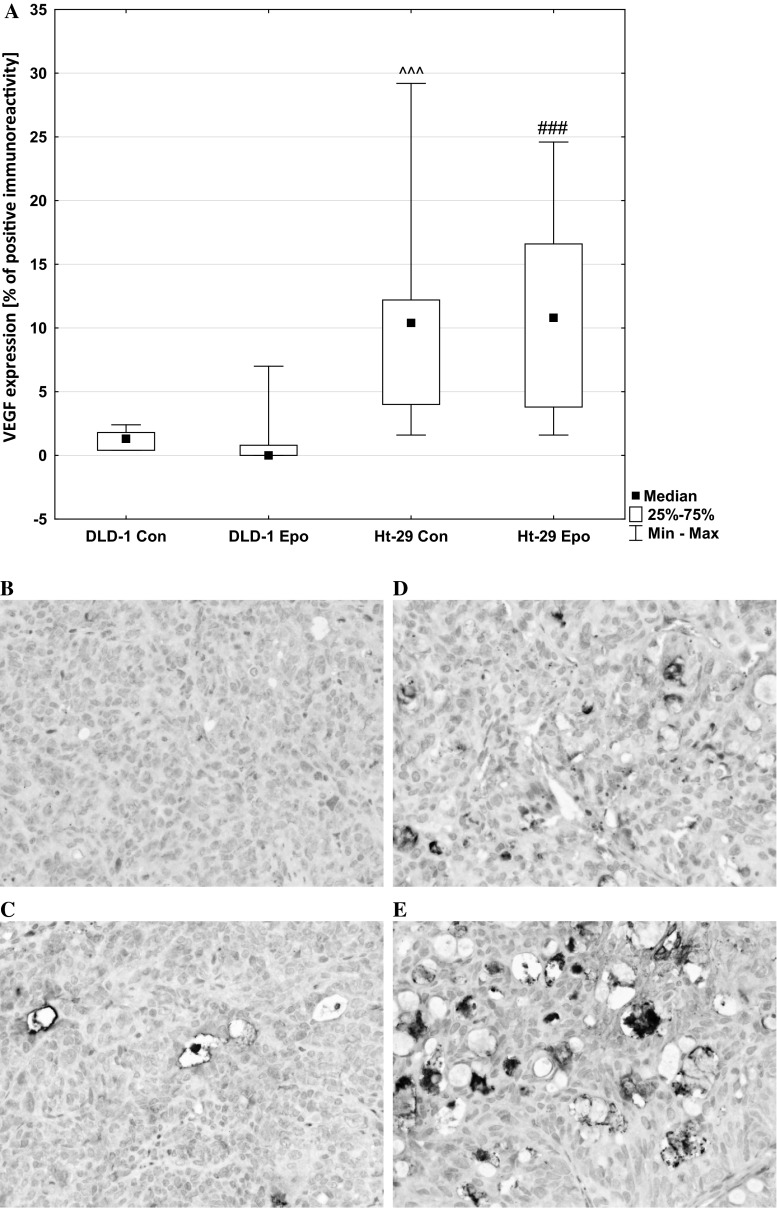
Positive expression of VEGF in the blood vessels of tumors: **a** a box-and-whisker plot of percent of VEGF expression in DLD-1 and Ht-29 tumor xenografts. Results are presented as medians (minimum–maximum), *n* = 10, ^^^*p* < 0.001 (Con in DLD-1 vs. Con in Ht-29 xenografts), ###*p* < 0.001 (Epo in DLD-1 vs. Epo in Ht-29 xenografts). **b** expression in control DLD-1 xenografts; **c** expression in DLD-1 xenografts receiving Epo; **d** expression in control Ht-29 xenografts; **e** expression in Ht-29 xenografts receiving Epo (H&E staining; *magnification* ×400)

Angiogenesis is essential for tumor growth and metastatic activity. This process is regulated by a variety of angiogenic molecules, predominantly driven by VEGF, a proangiogenic growth factor expressed by many solid cancers. For this purpose, we examined the effects of erythropoietin on VEGF and Flt-1 expression. Analysis of VEGF expression revealed no reaction in tumor cells. Only in two Ht-29 xenografts (10 % of all cases) weak, positive, cytoplasmic reaction was observed in individual cells of stromal tumor. In contrast, its positive expression was observed in the tumor vasculature. Mann–Whitney non-parametric *U* test confirmed the significant differences in tumor vessels VEGF expression in both control (*p* < 0.001) and erythropoietin-treated DLD-1 xenografts compared with Ht-29 xenografts (*p* < 0.001) (Fig. 9). In tumor vessels, VEGF expression was associated with moderately differentiated colorectal adenocarcinomas. The median, minimum, and maximum of VEGF are presented in Fig. 9a.

**Fig. 10:**
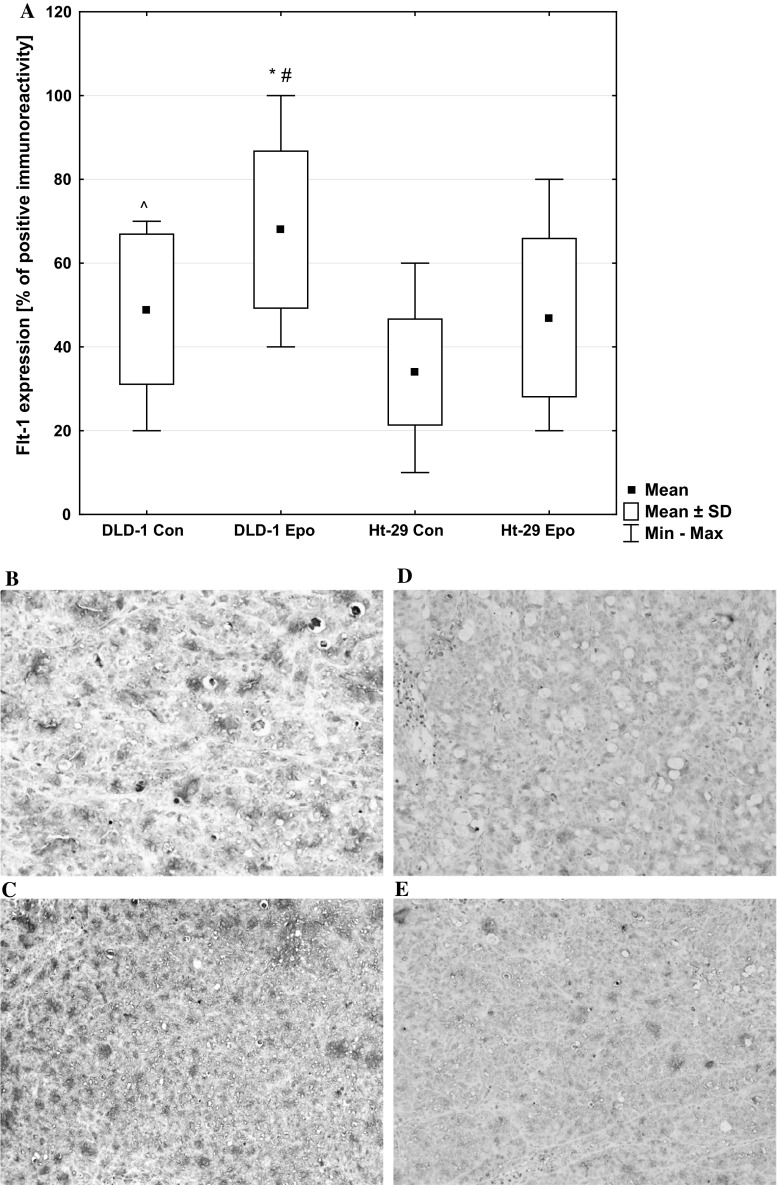
Positive expression of Flt-1 in membrane and cytoplasm of colon cancer xenografts: **a** a box-and-whisker plot of percent of Flt-1 expression in DLD-1 and Ht-29 tumor xenografts. Results are presented as means ± SDs, *n* = 10, *p < 0.05 (Con vs. Epo in DLD-1 xenografts), ^*p* < 0.05 (Con in DLD-1 vs. Con in Ht-29 xenografts), #*p* < 0.05 (Epo in DLD-1 vs. Epo in Ht-29 xenografts). **b** expression in control DLD-1 xenografts; **c** expression in DLD-1 xenografts receiving Epo; **d** expression in control Ht-29 xenografts; **e** expression in Ht-29 xenografts receiving Epo (H&E staining; *magnification* ×400)

Mixed (membranous and cytoplasmic) immunoreactivity for Flt-1 was observed in all positive slides. The means and standard deviations of Flt-1 are presented in Fig. 10. Most sections stained more intensely for Flt-1 than for VEGF. The two-factor analysis of variance showed a significant main effect for the group (control xenografts or xenografts receiving Epo) factor (*p* < 0.01), a significant main effect for the cell line factor (*p* < 0.01), but the interaction between group and cell line was not significant (*p* = 0.585). It also showed significant differences in Flt-1 expression in DLD-1 and Ht-29 xenografts treated with Epo (*p* < 0.05). The Ht-29 xenografts indicated a considerable level of Flt-1 in control and the erythropoietin-treated group (Fig. 10). A statistically significant positive correlation was observed between EpoR and Flt-1 expression in DLD-1 xenografts treated with Epo (*r* = 0.688, *p* = 0.028), as well as between VEGF and Flt-1 expression in DLD-1 xenografts treated with Epo (*r* = 0.734, *p* = 0.016).

**Fig. 11:**
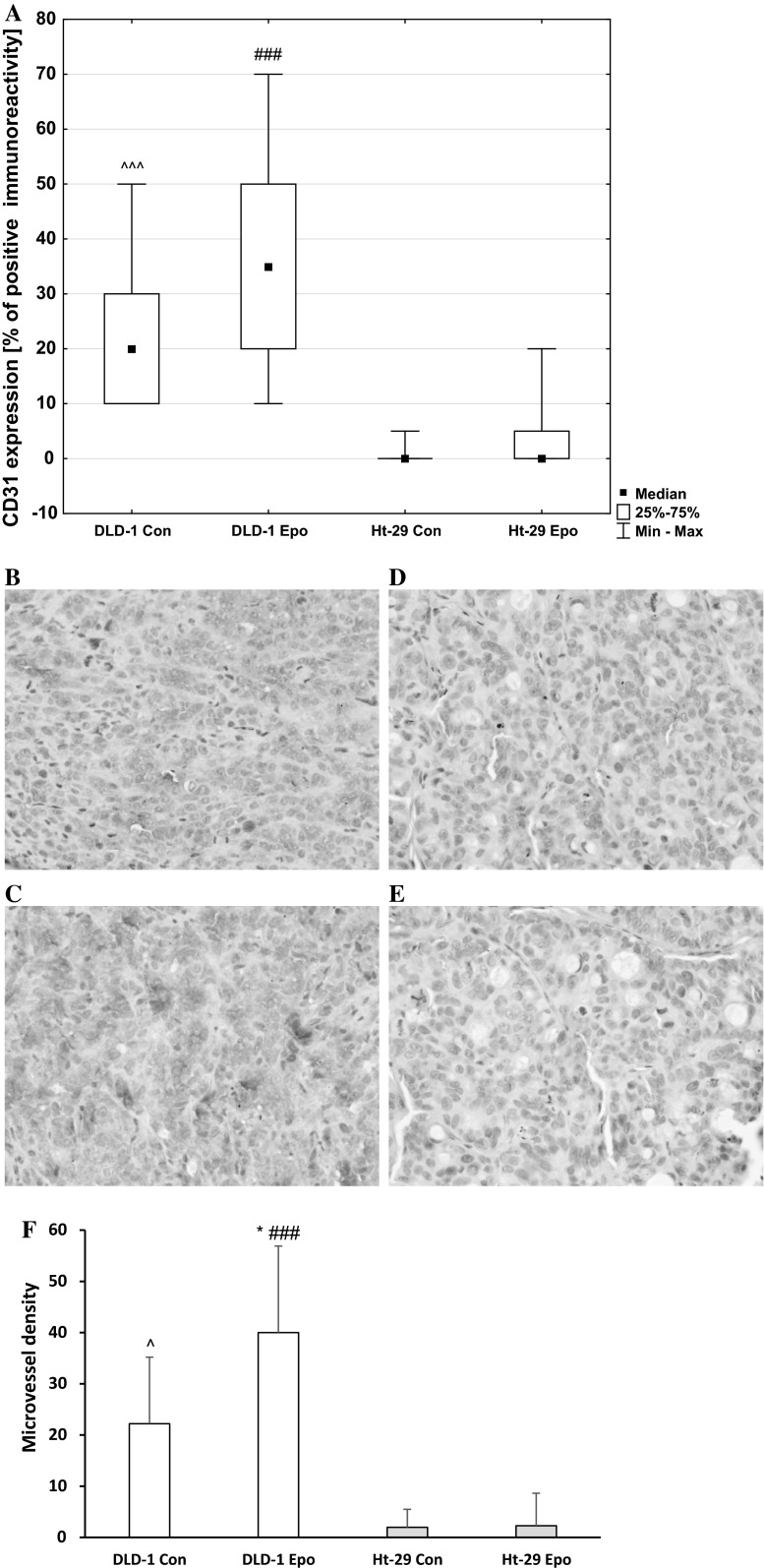
Positive expression of CD31 in membrane and cytoplasm of colon cancer xenografts: **a** a box-and-whisker plot of percent of CD31 expression in DLD-1 and Ht-29 tumor xenografts. Results are presented as medians (minimum–maximum), *n* = 10, ^^^*p* < 0.001 (Con in DLD-1 vs. Con in Ht-29 xenografts), ###*p* < 0.001 (Epo in DLD-1 vs. Epo in Ht-29 xenografts). **b** expression in control DLD-1 xenografts; **c** expression in DLD-1 xenografts receiving Epo; **d** expression in control Ht-29 xenografts; **e** expression in Ht-29 xenografts receiving Epo; **f** microvessel density in DLD-1 and Ht-29 xenografts, *p < 0.05 (Con in DLD-1 vs. Epo in DLD-1 xenografts), ^*p* < 0.05 (Con in DLD-1 vs. Con in Ht-29 xenografts), ###*p* < 0.001 (Epo in DLD-1 vs. Epo in Ht-29 xenografts). (H&E staining; *magnification* ×400)

The CD31 protein is a known marker of normal and neoplastic vascularization. CD31 expression was observed mainly in DLD-1 xenografts and associated with poorly differentiated adenocarcinoma of the colon. Cytoplasmic and membrane reaction anti-CD31 staining prevailed in all positive slides. Positive immunoreactivity for CD31 was found in all DLD-1 xenografts: 10 (50 % of all cases) of these exhibited weak staining and 10 (50 % of all cases) exhibited strong staining. In Ht-29 xenografts, weak, positive immunoreactivity for CD31 was observed only in 3 animals (15 % of all cases). Mann–Whitney non-parametric *U* test confirmed the significant differences in CD31 expression in control DLD-1 xenografts compared with Ht-29 xenografts (*p* < 0.001), as well as in erythropoietin-treated DLD-1 xenografts compared with Ht-29 xenografts (*p* < 0.001) (Fig. 11). The median, minimum, and maximum of CD31 are presented in Fig. 11a.

The mean (SD) CD31- microvessel density (MVD) in the tumor specimens was 22.3 (13.02) in control DLD-1 xenografts, 40.0 (16.9) in erythropoietin treatment DLD-1 xenografts, 2.0 (3.5) in control Ht-29 xenografts and 2.3 (6.3) in Ht-29 xenografts receiving erythropoietin. Statistical analysis showed significant increase of tumor MVD in erythropoietin-treated DLD-1 xenografts compared with control DLD-1 xenografts (*p* < 0.05) and compared with Ht-29 xenografts (*p* < 0.001), as well as in control DLD-1 xenografts compared with Ht-29 xenografts (*p* < 0.005). The mean and SD of MVD are presented in Fig. 11 F. These results show that erythropoietin in EpoR-positive xenografts through increase of VEGF and Flt-1 expressions may contribute to tumor development by promoting angiogenesis. Noticeable increase of tumor microvessel density in erythropoietin-treated DLD-1 xenografts additionally confirms proangiogenic nature of Epo.

## Discussion

In this study, we examined the expression of EpoR and Akt in adenocarcinoma cells as well as EpoR, VEGF, Flt-1, and CD31 expression in xenografts. We found that EpoR is constitutively expressed in colon cancer cells, although its expression is dependent on cell line type. EpoR expression levels were significantly higher in DLD-1 cells compared with Ht-29 cells. Administration of Epo stimulated proliferation only in EpoR-positive DLD-1 cells, and also increased p-EpoR levels in DLD-1 and p-Akt levels in both DLD-1 and Ht-29 cells. We observed differences in tumor growth in DLD-1 and Ht-29 xenografts. We showed fast tumor growth in moderately differentiated grade Ht-29 colon cancer xenografts. Subcutaneous administration of Epo led to enhancement of carcinogenesis through increase of EpoR, Flt-1, VEGF, and CD31 expression. Thus, treatment with Epo stimulated the growth of EpoR-positive tumor of DLD-1 xenografts by promoting angiogenesis. Erythropoietin via EpoR potentiated proliferation by increased levels of p-Akt in DLD-1 cells in vitro.

On the other hand, necrosis was observed in all DLD-1 xenografts. These results suggest that apart from stimulating cell proliferation phosphorylated Akt can also promote cell death. The antiapoptotic activity of Akt has been well documented, through a number of mechanisms, including inhibition of caspase 9, Bad, and GSK-3β, as well as induction of mitochondrial hexokinase and nuclear factor kappa B-dependent antiapoptotic gene expression [30, 31]. Despite this, several recent reports show an emerging role for Akt as a ‘death kinase’ and a key regulator of programmed necrosis, including neuronal cell types [32].

These results are consistent with the notion that Epo is the cause of negative effects on oncologic outcome by enhancing tumor growth. Gombos et al. indicate that Epo and EpoR showed enhanced expression in areas adjacent to ischemia/necrosis in colorectal cancer. Increasing expression of Epo and EpoR along the colonic neoplastic sequence and further increase in ischemic/necrotic areas indicates that the Epo signaling pathway is an important component in colon carcinogenesis [12].

The available literature data suggest that Epo/EpoR may play an important role in tumor progression [5, 6, 33]. Although Epo and its receptor EpoR were originally believed to have an exclusive role in erythropoiesis, they are also expressed in many non-hematopoietic cells including certain tumor tissue. Their presence has been demonstrated in breast, cervical, endometrial, gastric, and colon cancer [7, 8, 21].

Due to limited availability of live primary cells from recessions or biopsies, we investigated EpoR expression on DLD-1 and Ht-29 colon cancer cell lines. The results of our previous research and data from the literature indicate that the DLD-1 line contains the EpoR gene and protein, and histologically is the most similar to a primary tumor [22]. Using real-time RT-PCR and confocal microscopy, we confirmed the existence of EpoR in DLD-1 but little/no expression in Ht-29. In the case of Ht-29, our results are comparable with the report that EpoR is undetectable in Ht-29 cultures and is a negative control for the EpoR gene [22].

Our in vivo studies with nude mice confirmed the in vitro results. Also, we observed a significant difference in EpoR expression in DLD-1 xenografts treated with Epo compared with Ht-29 xenografts. Our results are contrary to evidence of documented EpoR expression in Ht-29 [34]. Our data also conflict with the hypothesis of the absence of functionally expressed EpoR in the DLD-1 cell line [35]. Incubation with Epo did not change EpoR mRNA expression in DLD-1 and Ht-29 cells, but increased p-EpoR levels in DLD-1 cells. Thus, in EpoR-positive colon cancer cell line, administration of agonist caused an increase in active receptor levels. In turn, in the EpoR-negative line, the exogenous agonist did not cause any changes in EpoR mRNA expression as well as in the level of active EpoR. Some investigators also observed negative regulatory pathways that lead to the termination of intracellular signals after the incubation of cells with Epo [35]. As the authors suggested, these negative signaling events are caused by receptor dephosphorylation by the phosphotyrosine-phosphatase SHP1, internalization, and degradation of the activated receptor.

We also evaluated whether stimulation with exogenous Epo induces cell proliferation. We found that Epo induced proliferation of DLD-1 cells, whereas the addition of Epo to Ht-29 has no effect on their proliferation. Our results indicate that response of tumor cells to Epo is variable and depends on the presence of EpoR. Some previous studies show that Epo treatment can increase tumor cell numbers in vitro [6, 11, 25], whereas others found no effect [36]. These observations suggest that the effects of Epo on a particular kind of tumor may not be consistent between cell lines. The cause of these conflicting results may be connected with the different sensitivities of the used tumor cell lines.

Our results suggest that Epo possesses a different effect on DLD-1 and Ht-29 cells, which are possibly associated with lack or minimal presence of EpoR receptors on the Ht-29 tumor cell line. Results of our study show that EpoR is located not only on the cell surface but also in the cytoplasm. This observation was confirmed by using two independent methods, such us confocal microscopy for colon cancer cells as well as immunohistochemistry for ex vivo samples. For many years it was shown that erythropoietin receptor is located on the surface of erythroid progenitor cells [37]. Acs et al. using immunohistochemistry found that EpoR were present in tumor cells but were absent from the surrounding normal breast tissue. This is important, because it may be possible to use a tumor’s EpoR to target a drug to a tumor and not damage the surrounding tissues. This concept appears to have potential for future exploration [8].

Activation of the EpoR-associated Akt kinase occurs immediately after Epo binding and has been documented in various types of cancer [38, 39]. Most of data on Epo-induced signaling response indicated higher levels of p-Akt, a molecule which phosphorylates serine/threonine kinase—mTOR involved in numerous cellular processes associated with growth, proliferation, infiltration capacity, and in some cells with apoptosis. In the current study, incubation of DLD-1 with Epo induced an increase in p-Akt levels in DLD-1. Our results clearly indicate that increased cell proliferation after Epo/EpoR activation is related with the PI3K/Akt signaling pathway. To our surprise, we observed an Epo-induced increase in p-Akt levels also in Ht-29, the cell line with a low expression of EpoR. This finding suggests that levels of p-Akt depend on different mechanisms responsible for not only EpoR activation. The beta-common receptor, which is a shared receptor subunit for interleukin-3, interleukin-5, GM-CSF, and is also activated by erythropoietin, has been demonstrated to initiate the Akt signaling pathway [40]. In lymphoma cells, this receptor is involved in the promotion of cell growth, proliferation, and survival. Erythropoietin appears to promote cell survival in an EpoR-independent mechanism in cells with no/low expression of EpoR. In our study, Akt mRNA expression was significantly reduced after incubation of Ht-29 cells with erythropoietin. We suppose that the cause of this phenomenon is the occurrence of a negative feedback loop between the level of phosphorylated Akt and expression of Akt mRNA in cancer cells. Rozengurt reported that mTORC1/S6 K axis not only promotes growth-promoting signaling but also mediates potent negative feedback loops that restrain upstream signaling through tyrosine kinase receptors in both normal and oncogene-transformed cells. Suppression of these feedback loops by inhibitors of mTORC1/S6K causes compensatory over-activation of upstream signaling nodes, including PI3 K, Akt, and ERK [41].

To confirm the results of in vitro studies, we used an in vivo cancer model. 4-week-old mice Cby.Cg-Foxn1nu/J were inoculated with DLD-1 and Ht-29 colon cancer cells and treated with Epo. Erythropoietin in a clinical dose of 600 IU/kg significantly accelerated tumor growth only in DLD-1 xenografts. We also examined 4 weeks dynamic of tumor growth and we found that Ht-29 xenografts tumors were larger than DLD-1 but were less responsive to Epo treatment. Our study demonstrated a direct relationship between Epo exposure and tumor growth in DLD-1 xenografts. We observed similar results in our in vitro studies, in which only DLD-1 cells increased proliferation after Epo incubation. Thus, the presence of EpoR in colon cells seems to play a significant role in the response to Epo. Recent studies carried out on xenografts of human colon cancer clearly proved a significant role of Epo in the formation of recurrence after radical surgery. It was found that in a group of Epo-treated animals, the ratio of recurrence and tumor volume was greater in animals receiving erythropoietin compared with the control group [17].

One way, by which Epo could trigger tumor growth, is its direct effect on the tumor cells via the EpoR. Erythropoietin has been reported to stimulate tumor growth in EpoR-expressing tumors such as breast, cervical, endometrial, gastric, and colon cancer. In an in vivo study, Arcasoy et al. showed that blocking EpoR stopped tumor growth in a breast cancer rat model [42]. Yasuda et al. found that blocking Epo function can inhibit the progression of stomach choriocarcinoma, melanoma, ovarian, and uterine tumors [9, 43]. Here immunohistochemical analysis demonstrated weak levels of EpoR expression in Ht-29 cells in 95 % of positive tumors, which is a possible reason for the poor response to Epo exposure. Contrary, the high level of EpoR expression found in DLD-1 tumors was linked directly with tumor size after Epo administration.

Another presumed mechanism by which Epo could promote tumor growth is angiogenesis. EpoR expression in tumor vascular endothelium suggests that Epo may affect the tumor microenvironment by enhancing vessel formation [10]. Other studies, which found the presence of EpoR on endothelial cells of tumor capillaries as well as reduction of the capillary network by blocking EpoR in vitro, are another confirmation of the participation of Epo in tumor vascularization [43]. Jaquet et al. found the angiogenic potential of Epo to be similar to that of vascular endothelial growth factor (VEGF) when stimulating human adult myocardial endothelial cells. It is also known that tumor vascularization plays a key role in tumor growth and progression, as well as metastasis [44]. The solid tumors, including tumors of the colon, have the ability to produce and secrete factors such as vascular endothelial growth factor (VEGF) and fibroblast growth factor (FGF), whose main function is angiogenesis. Platelet endothelial cell adhesion molecule-1 (CD31/PECAM-1) plays an important role in angiogenesis. During angiogenesis CD31 participates in adhesive and signaling pathway required for the endothelial cells movement and their following organization into vascular tubes [45]. We examined the expression of VEGF, Flt-1 (VEGF-receptor), and CD31 in DLD-1 and Ht-29 xenografts using immunohistochemical staining. Epo administration stimulated Flt-1 expression in DLD-1 xenografts. In these animals, we observed a statistically significant positive correlation between EpoR and Flt-1 expression, as well as between VEGF and Flt-1.

In this report, we demonstrate that Epo stimulates angiogenesis in DLD-1 xenografts. Our results support the notion that angiogenesis activation is the mechanism by which Epo promotes tumor growth. This is in agreement with other authors who showed that the erythropoietin/erythropoietin-receptor system is involved in angiogenesis in human hepatocellular carcinoma [10]. Our preliminary study on rats with chemically induced tumors also showed that Epo stimulates angiogenesis, and thus contributes to better blood circulation and oxygenation of the tumor cells. We also found that ‘poorly differentiated’ DLD-1 has a higher degree of vascularization than ‘moderately’ Ht-29. EpoR expression in colon cancer cells increased in parallel with malignancy grade and was highly correlated with the range of angiogenesis.

In conclusion, these results show that both EpoR-positive and EpoR-negative cancer cells could be regulated by exogenous Epo. In the EpoR-positive cells, Epo treatment can enhance carcinogenesis through increase of EpoR and Flt-1 expression, and thereby contributed to tumor development. Our results not only expand the existing knowledge but also resolve the controversy over using Epo in colon cancer patients. Although the clinical benefits of Epo therapy appear to be significant, data from the present study suggest that this hormone should not be used for treatment of anemia in patients with colon cancer. Moreover, clinicians should also be careful while using Epo with other patients carrying both EpoR-positive and EpoR-negative tumors.
